# Integrated analysis of multi-omics data reveals T cell exhaustion in sepsis

**DOI:** 10.3389/fimmu.2023.1110070

**Published:** 2023-04-03

**Authors:** Qiaoke Li, Mingze Sun, Qi Zhou, Yulong Li, Jinmei Xu, Hong Fan

**Affiliations:** ^1^ Department of Respiratory and Critical Care Medicine, West China Hospital/West China School of Medicine, Sichuan University, Chengdu, China; ^2^ Department of Intensive Care Unit, Sichuan Provincial Crops Hospital of Chinese People’s Armed Police Force, Leshan, China; ^3^ Department of Oncology, Jiang’an Hospital of Traditional Chinese Medicine, Yibin, China

**Keywords:** septic shock, acute respiratory distress syndrome (ARDS), receiver operating characteristic (ROC), ppara peroxisome proliferator-activated receptor α, Tesaglitazar

## Abstract

**Background:**

Sepsis is a heterogeneous disease, therefore the single-gene-based biomarker is not sufficient to fully understand the disease. Higher-level biomarkers need to be explored to identify important pathways related to sepsis and evaluate their clinical significance.

**Methods:**

Gene Set Enrichment Analysis (GSEA) was used to analyze the sepsis transcriptome to obtain the pathway-level expression. Limma was used to identify differentially expressed pathways. Tumor IMmune Estimation Resource (TIMER) was applied to estimate immune cell abundance. The Spearman correlation coefficient was used to find the relationships between pathways and immune cell abundance. Methylation and single-cell transcriptome data were also employed to identify important pathway genes. Log-rank test was performed to test the prognostic significance of pathways for patient survival probability. DSigDB was used to mine candidate drugs based on pathways. PyMol was used for 3-D structure visualization. LigPlot was used to plot the 2-D pose view for receptor-ligand interaction.

**Results:**

Eighty-four KEGG pathways were differentially expressed in sepsis patients compared to healthy controls. Of those, 10 pathways were associated with 28-day survival. Some pathways were significantly correlated with immune cell abundance and five pathways could be used to distinguish between systemic inflammatory response syndrome (SIRS), bacterial sepsis, and viral sepsis with Area Under the Curve (AUC) above 0.80. Seven related drugs were screened using survival-related pathways.

**Conclusion:**

Sepsis-related pathways can be utilized for disease subtyping, diagnosis, prognosis, and drug screening.

## Introduction

Sepsis is the major cause of morbidity and death in the intensive care unit (ICU) (1). Sepsis-1 defined sepsis as a host’s systemic inflammatory response syndrome (SIRS) to infection, and the clinical criterion was suspected infection plus SIRS (2). There are many causes of sepsis, such as bacterial infections, influenza, pneumonia, and food poisoning (3). ICUs are rare recourses worldwide. The definition was then revised and validated with new clinical criteria. The third international consensus on the definition of sepsis and septic shock defined sepsis as a syndrome of physiological, pathological, and biochemical abnormalities of the body induced by infection (4). Sepsis-3 criteria defined sepsis as “Life-threatening organ dysfunction caused by a dysregulated host response to infection” (5). Inflammation resulting from an overreaction to an infectious agent can induce fatal diseases such as hypotensive septic shock or septic shock, which have a high mortality rate (6). Currently, there is a large amount of transcriptomic data on sepsis in public databases, however, the molecular mechanisms of sepsis remain unclear. The heterogeneity of sepsis conditions and diverse sources of infection may contribute to this result (7). Conventional single-gene-based approaches are ineffective in distinguishing sepsis from traditional markers such as procalcitonin (PCT) and interleukin-8 (IL-8) (8). Recombinant human activated protein C (rhAPC) is currently the only drug approved by the U.S. Food and Drug Administration (FDA) for the treatment of severe sepsis. Therefore, it is necessary to develop molecular diagnostic markers based on a higher level. We propose to use the KEGG pathway as a marker in the diagnosis, classification, treatment, and prognosis of sepsis and provide potential therapeutic drugs accordingly. This method provides a novel idea from disease mechanism research to clinical translation. Here, we identify KEGG pathways associated with sepsis, survival, immune cells, and infection types, and discover potential disease-related drugs to provide important information for follow-up research.

## Materials and methods

### Datasets download

The sepsis-related transcriptome datasets were downloaded from the NCBI GEO database. GSE185263 was obtained from the next-generation sequencing platform Illumina HiSeq 2500, with a total of 392 whole blood samples, including 44 healthy controls and 392 sepsis patients. GSE65682 from the gene chip platform Affymetrix Human Genome U219 Array contains a total of 802 whole blood samples from sepsis patients. GSE63990 was derived from the Affymetrix Human Genome U133A 2.0 Array with a total of 273 whole blood samples, of which 88 were systemic inflammatory response syndrome (SIRS), 115 were viral acute respiratory infections, and 70 were bacterial acute respiratory infections. Cell type level RNA-Seq dataset GSE133822 profiled CD4^+^, CD8^+^, and CD14^+^ cells from 231 sepsis and critically ill patients. GSE40012 contains time serial blood transcriptome data from 8 influenza A pneumonia patients, 16 bacterial pneumonia patients, and 3 mixed bacterial and influenza A pneumonia patients. Sepsis methylation dataset GSE138074, which contains 11 healthy donors, 4 SIRS, and 14 sepsis, was downloaded from NCBI GEO. Single-cell data was retrieved from Human Universal Single Cell Hub and Broad Institute Single Cell Portal.

### Transcriptome and methylome data processing

The expression values in the expression matrix were converted to ranks, and the GSVA package in R (v4.0.3) was used to convert the gene expression matrix to pathway enrichment score (ES) (9). Differential KEGG pathway analysis was performed using the limma package, and the P value was set at 0.01. Pathway expression-based survival analysis was performed using the survival package, and the P value was set at 0.05. Immune cell abundance was analyzed using the TIMER online analysis tool (http://timer.comp-genomics.org/) (10). Pathway-based drug screening was performed by using the DSigDB database (http://dsigdb.tanlab.org/DSigDBv1.0/) (11). Methylation data were analyzed in the R ChAMP package according to the Illumina BeadChips analysis pipeline with P <0.05 (12). Functional enrichment of differentially methylated genes was performed in the R clusterProfiler package with adjusted P <0.05 (13). 3D structure visualization for the result of molecular docking was analyzed using PyMol 1.7.4.5 Edu. A 2D pose view of ligand and receptor interactions was drawn using LigPlot v2.2.5 (14). ROC curves were drawn using the pROC package. Survival curves were plotted by the survminer package.

### Statistical analysis

Student t test was used for the comparison of mean differences between two groups of data. Analysis of variance (ANOVA) was used to compare the mean difference between multiple groups of data. The correlation between two variables was analyzed by using Spearman and Pearson correlation coefficient analysis for variables with the same and different units, respectively. Differences between survival curves were analyzed by using the log-rank test.

## Results

### Analysis of KEGG pathway expression changes

**Figure 1:**
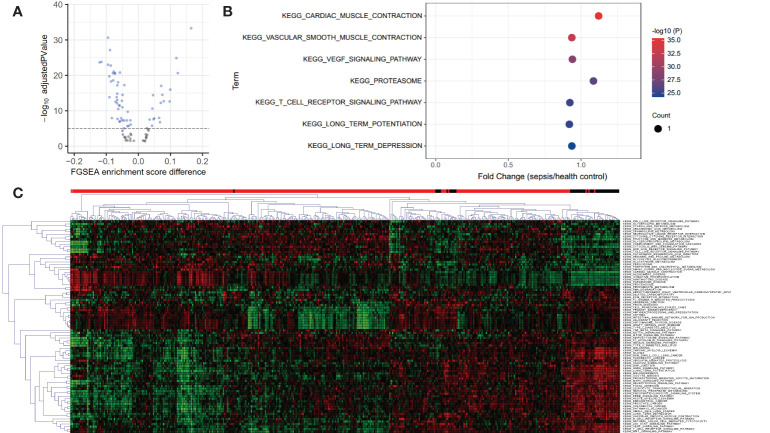
Volcano, bubble plots, and Clustering heatmap of the KEGG pathway differentially expressed in blood from healthy controls and septic patients in dataset GSE185263. **(A)** Volcano plot for 84 KEGG pathways altered in sepsis patients compared with healthy controls at adjusted P < 0.05. **(B)** Bubble plot for the top 7 most significant differentially expressed pathways. **(C)** Clustering heatmap based on 84 differential KEGG pathways reveals the heterogeneity of sepsis. The above bar indicates sample status, red: sepsis and black: healthy controls.

A total of 84 KEGG pathways were altered in dataset GSE185263 sepsis patients compared with healthy controls at adjusted P < 0.05 (**Figure 1A**; **Table S1**). **Figure 1B** shows the pathways with the most significant P value are cardiac muscle contraction (up-regulated), vascular smooth muscle contraction (down-regulated), vascular endothelial growth factor (VEGF) signaling pathway (down-regulated), proteasome (up-regulated), and T cell receptor (TCR) signaling pathway (down-regulated). The down-regulated pathways with the highest fold change are long term potentiation (LTP), TCR signaling, and vascular smooth muscle contraction. The most up-regulated pathways are proteasome, cytosolic DNA sensing pathway, and cardiac muscle contraction. Clustering analysis showed that the KEGG pathways had different expression patterns in sepsis and normal subjects, and some pathways also had different expression patterns among sepsis patients (**Figure 1C**). This may be related to the heterogeneity of sepsis itself, and also suggests the existence of different subtypes.

### Differential KEGG pathways were also identified in blood single-cell datasets

**Figure 2:**
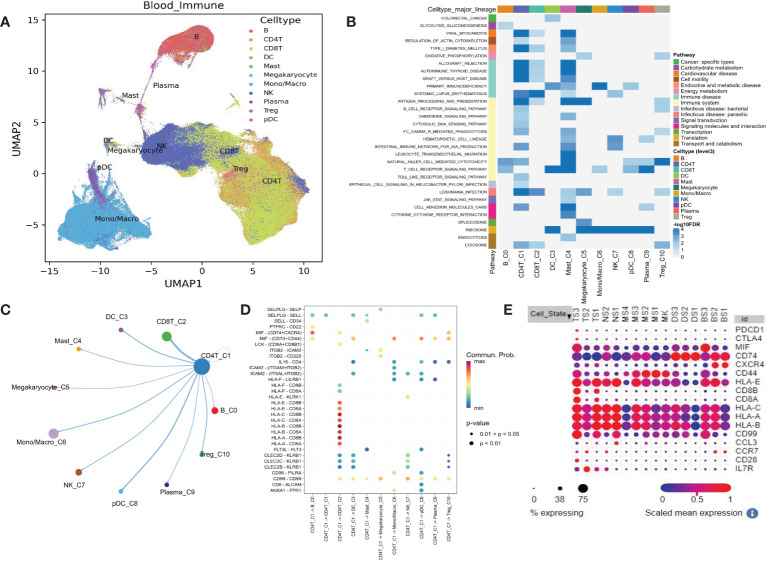
Single-cell transcriptome analysis shows the KEGG pathway differentially expressed in blood from healthy controls and potential mechanisms. **(A)** UMAP plot for the 11 immune cell clusters. **(B)** Heatmap shows some down-regulated pathways. **(C)** Circular plot for cell communications between CD4^+^ T cell and other 10 immune cells. **(D)** All the significant ligand-receptor pairs that contribute to the signaling sending from CD4^+^ T cell and other 10 immune cells. **(E)** The expression dot plot shows two sub-clusters TS3 and TS2 associated with T cell exhaustion. TS, T cell subclusters; NS, NK cell subclusters; MS, monocyte/macrophage subclusters; MK, megakaryocyte subclusters; DS, dendritic cell (DC) subclusters; BS, B cell subclusters.

We confirmed that some of the most significant pathways were also presented in the blood single-cell dataset, indicating the cellular source of expression variation. Uniform Manifold Approximation and Projection (UMAP) dimension reduction analysis showed that the 483286 blood cells were organized into 10 major clusters according to their gene expression patterns (**Figure 2A**). Tregs are neighbored with CD4^+^ and CD8^+^ T cells, indicating their potential functional connections. Further KEGG pathways expression was analyzed in different single-cell clusters, and several pathways were recurrently identified as those in the bulk transcriptome analysis. These pathways involve the immune system in CD4^+^ T cells, such as antigen processing and presentation, B cell receptor signaling pathway, Fc gamma R mediated phagocytosis, intestinal immune network for IgA production, and TCR signaling pathway (**Figure 2B**). Cell-cell interaction analysis showed that CD4^+^ T cell closely interacted with CD8^+^ T cell by MIF, LCK, and HLA genes (**Figures 2C, D**). Dot plot of these genes in sepsis single-cell data revealed the CD8^+^ T cell exhaustion subcluster (**Figure 2E**, TS3 with high CD8^+^ T cell markers CD8a, CD8b, and exhaustion markers PDCD1 and CTLA4). CD4^+^ T cell exhaustion subcluster was also observed (**Figure 2E**, TS2 with high CD4^+^ T cell marker IL7R, and exhaustion marker CTLA4). Therefore, T cell exhaustion is present in sepsis.

### Relationship between KEGG pathway expression and 28-day survival rate

**Figure 3:**
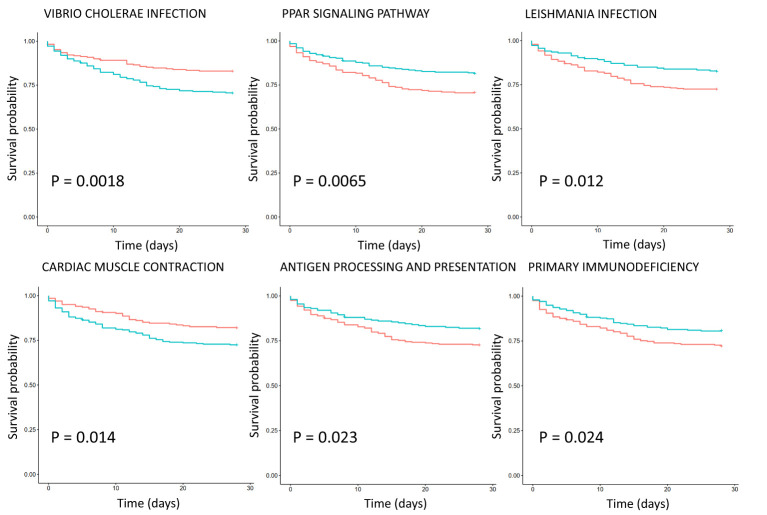
Survival analysis of KEGG pathway expression in severe sepsis patients based on dataset GSE65682. The red curve represents the pathway with low expression, and the green curve represents the pathway with high expression. The P value is obtained according to the log-rank test, and the top 6 pathways with the most significant P value are displayed.

Survival analysis of critically ill patient dataset GSE65682 showed that the KEGG pathways are associated with survival (**Figure 3**). Eight KEGG pathways expression were positively correlated with 28-day survival, and 2 were negatively correlated. Positively related pathways include antigen processing and presentation (down-regulation), chronic myeloid leukemia (CML, down-regulation), cytokine-cytokine receptor interaction (up-regulation), cytosolic DNA sensing pathway (up-regulation), *Leishmania* infection, PPAR signaling pathway, primary immunodeficiency (down-regulation) and progesterone mediated oocyte maturation. Negatively related pathways include cardiac muscle contraction and *Vibrio cholerae* infection. Among them, *Vibrio cholerae* infection and PPAR signaling pathway are the two most significant pathways, with P values less than 0.01.

### Analysis of the relationship between the KEGG pathway and immune cell abundance

**Figure 4:**
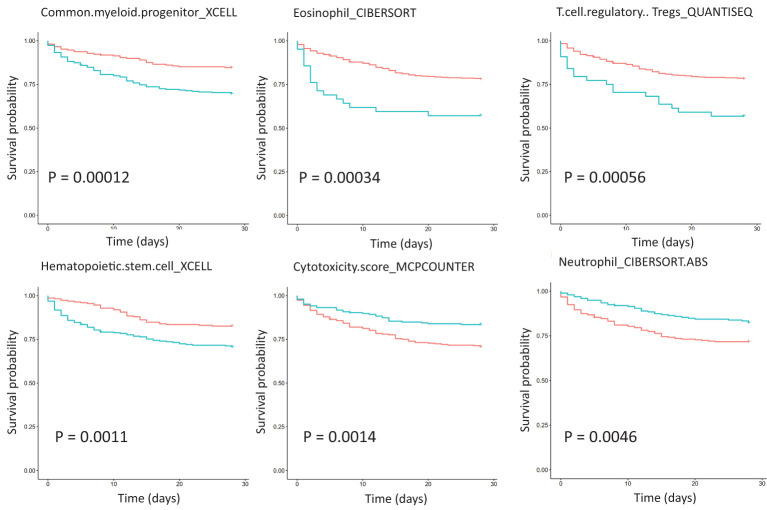
Survival analysis of immune cell abundance in critically ill patients with sepsis based on dataset GSE65682, showing the top six immune cell types with the most significant P values. The red curve represents the low expression of the pathway, the green curve represents the high expression of the pathway, and the P value is obtained according to the log-rank test.

Survival analysis of critically ill patient dataset GSE65682 showed that immune cell abundance was associated with survival (**Figure 4**). Common myeloid progenitor, eosinophil, regulatory T cells (Tregs), hematopoietic stem cells, NK cells, and 28-day survival were negatively correlated. Plasmacytoid dendritic cell, neutrophil, mast cell activated, and 28-day survival were positively correlated. Furthermore, total macrophages were positively correlated with 28-day survival, while non-activated M0 and polarized M1 macrophages were negatively correlated with 28-day survival. Correlation analysis of immune cell abundance and KEGG pathway expression showed that the KEGG pathway and specific immune cell abundance were significantly correlated (**Figure S1**). CD4^+^ T cells were positively correlated with 28-day survival, and their abundance had the highest correlation with the TCR signaling pathway (R =0.83, P <0.01), which is down-regulated in patients with sepsis. Centriocytes were positively correlated with 28-day survival, and their abundance was highly correlated with the Toll-like receptor (TLR) signaling pathway (R = 0.78, P < 0.01), which was up-regulated in sepsis patients. In addition, CD4 Th1 T cell abundance was negatively correlated with the insulin signaling pathway (R = -0.72, P < 0.01), with the highest negative correlation, and this pathway was down-regulated in sepsis patients. Differential expression of twenty-two immune cells between control and sepsis was shown in **Figure S2**.

**Figure 5:**
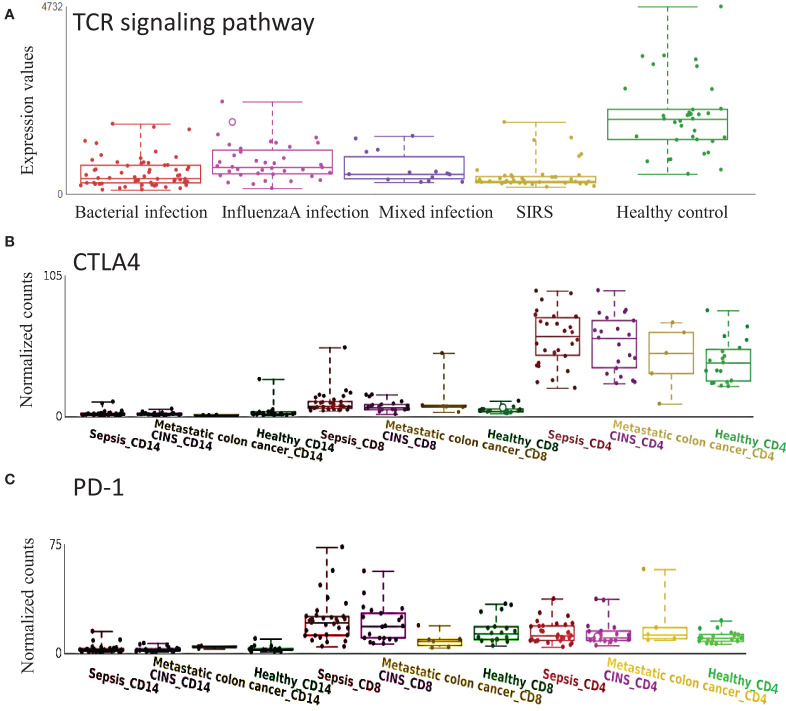
Expression of T cell receptor (TCR) signaling pathway and related genes are altered in sepsis. **(A)** TCR signaling pathway is down-regulated in sepsis with different etiology. **(B)** CTLA4 is mainly expressed on CD4^+^ T cells and is up-regulated in sepsis. **(C)** PD1 is mainly expressed on CD8^+^ T cells and is up-regulated in sepsis. CINS: critically ill non-infected subjects.

T cell exhaustion plays an important role in sepsis outcomes (15; (16). CTLA4 and PD-1, which may target TCR signaling and inhibit functional T cell activation, are well-known marker genes in T cell exhaustion (17). TCR signaling pathway was confirmed to be down-regulated in sepsis with various etiologies (**Figure 5A**). In the cell-type RNA-Seq dataset, we found that CTLA4 was mainly expressed on CD4^+^ T cells but not CD8^+^ T cells, and CTLA4 was significantly up-regulated in sepsis (**Figure 5B**). While PD-1 was mainly expressed on CD8^+^ T cell, which was significantly up-regulated in sepsis (**Figure 5C**). These data indicate the impaired adaptive immune effector cells, including CD4^+^ and CD8^+^ T cell exhaustion in sepsis.

### Identification of DNA methylation in sepsis blood samples

**Figure 6:**
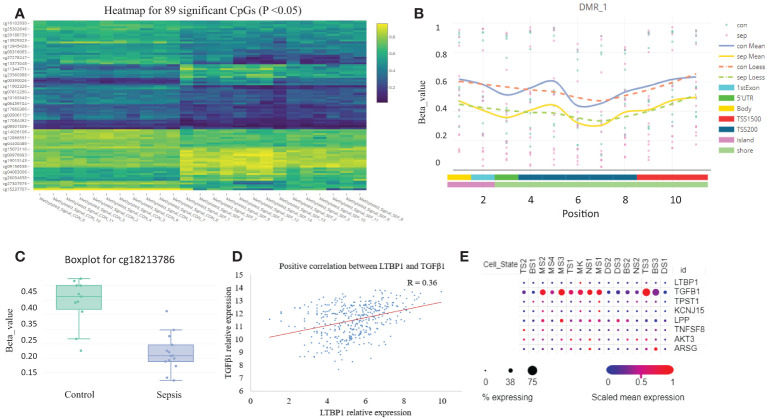
DNA methylation is altered in sepsis blood. **(A)** Heatmap plot for the 89 differentially methylated probes with adjusted P <0.05. **(B)** A representative plot for the hypomethylated region on chromosome 2. **(C)** Boxplot for probe cg18213786 which represents down-regulated methylation signal in gene LTBP1 (P =0.03). **(D)** The correlation scatter plot shows the positive expression relationship between LTBP1 and TGF-β1 (P =2E-11). **(E)** The expression dot plot shows cell type-specific expression of LTBP1 and TGF-β1. TS, T cell subclusters; NS, NK cell subclusters; MS, monocyte/macrophage subclusters; MK, megakaryocyte subclusters; DS, dendritic cell (DC) subclusters; BS, B cell subclusters.

We analyzed the sepsis DNA methylation dataset and found that no significant differential methylation probes, regions, or blocks were detected between SIRS and healthy control. A total of 89 significant differential methylation probes were identified between sepsis and control (**Figure 6A**; **Table S2**). Functional analysis showed that the corresponding genes were involved in GTPase activity regulation (P <0.05, **Figure S3**). TPST1, KCNJ15, and LPP were some of the most significant genes. LTBP1 was the only gene identified with two significant hypomethylation probes (**Figures 6B, C**). LTBP1 is associated with TGF-β signaling. Correlation analysis showed a positive correlation between LTBP1 and TGF-β1 (**Figure 6D**). Cell type gene expression analysis suggested that LTBP1 was mainly expressed on megakaryocytes (MK) and exhausted CD8^+^ T cells (TS3). TGF-β1 was mainly expressed in exhausted CD8^+^ T cells (TS3) and monocytes/macrophage (MS3) (**Figure 6E**). Further literature search of the 56 differentially methylated genes in PubMed identified that at least half of the genes are associated with T cells (**Table S2**). Thus, these results indicate that methylation regulates T cell function and exhaustion.

### Analysis of the relationship between the KEGG pathway, SIRS, and sepsis

**Figure 7:**
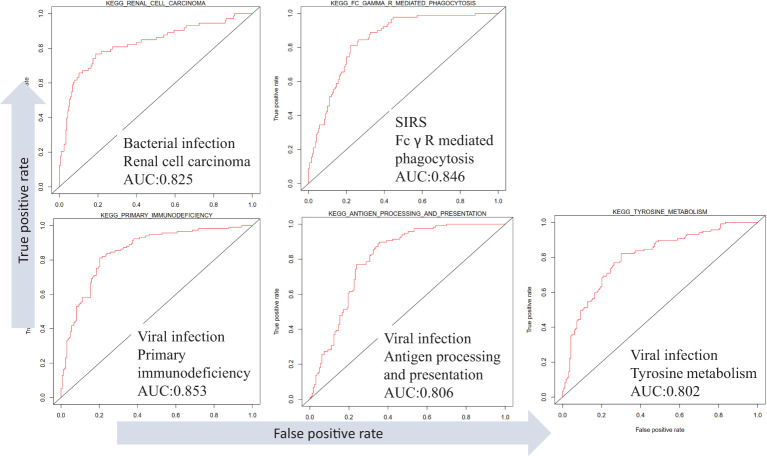
Differentiation of SIRS, bacterial and viral sepsis using KEGG pathway expression based on dataset GSE63990.

Different infection types in the dataset GSE63990 were distinguished, and the results showed that infection types were associated with different KEGG pathways (**Figure 7**). For example, high expression of renal cell carcinoma can distinguish bacterial infection (AUC =0.83). High expression of Fc gamma R mediated phagocytosis distinguishes non-infectious sepsis (AUC =0.85). High expression of primary immunodeficiency, antigen processing and presentation, and tyrosine metabolism distinguish viral infections (AUCs =0.85, 0.81, and 0.80). In addition, different infection types also have different low-expression pathways. For example, low expression of retinol metabolism can distinguish bacterial infection (AUC =0.82). Low expression of valine, leucine, and isoleucine degradation or peroxisome can distinguish non-infectious sepsis (AUCs =0.81 and 0.80). Low expression of cardiac muscle contraction or glycerophospholipid metabolism distinguishes viral infection (AUCs =0.84 and 0.81). Analysis of differential pathways between viral and bacterial sepsis (**Figure S4**) revealed that the five most significant pathways were primary immunodeficiency (up-regulated in viral sepsis, P =3E-27), antigen processing and presentation (up-regulated, P =3E-22), cell adhesion molecules (CAMS, up-regulated, P =3E-22), intestinal immune network for IgA production (up-regulated, P =5E-21) and cardiac muscle contraction (down-regulated, P =9E-21).

### Analysis of the relationship between T cell subsets and sepsis infection types

**Figure 8:**
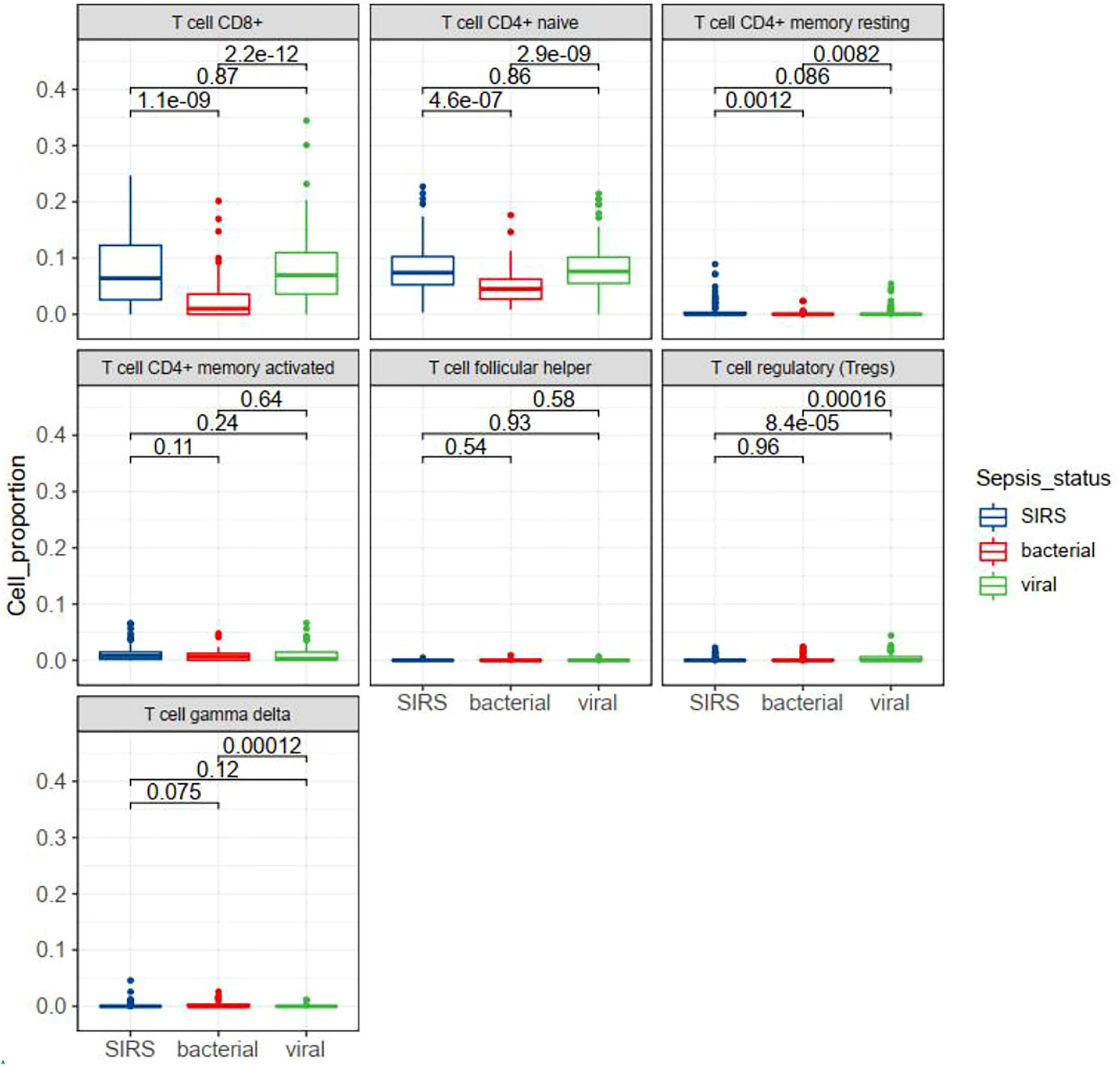
Box plots showing the differential expression of seven T cell subsets in different status of sepsis. SIRS, systemic inflammatory response syndrome.

T cell subsets in sepsis with different infection status were examined in GSE63990. By TIMER analysis we found that some subsets of T cells were differentially expressed between bacterial and viral sepsis. Among the seven T cell subsets, five cell subsets were differentially expressed in viral sepsis compared to bacterial sepsis, including up-regulated CD8^+^ T cell, naïve CD4^+^ T cell, resting memory CD4^+^ T cell, Tregs, and down-regulated gamma-delta T cell (**Figure 8**). To explore T cell subsets during the septic process, we compared their changes between alive and dead patients in GSE65682. We found that naive CD4^+^ T cells and gamma-delta T cells were down-regulated in the dead patients (**Figure S5A**). To be more infection-type specific, we analyzed GSE40012 which contains time serial transcriptome data of critically ill patients with bacterial and viral infections. We found that gamma-delta T cell was down-regulated in bacterial septic process (**Figure S5B**), while Tregs was up-regulated in viral septic process (**Figure S5C**, day 1 *vs*. day 5). To examine the effect of secondary infection on T cell subsets, we compared the T cells of patients with mixed infection and single infection. We found that Tregs were up-regulated in the mixed infection patients compared to single infection patients (**Figure S5D**), indicating the possible roles of Tregs in the secondary infection. We also performed multivariate analysis by Cox proportional hazards model to check the possible effect of T cell subsets on the prognosis of patients with sepsis. In the multivariate model, we observed that nearly all the original KEGG pathways were still prognostic for patient survival (**Table S3**). In sum, T cell subsets may play different roles in bacterial and viral sepsis.

### Screening of drugs for sepsis treatment based on KEGG pathways

**Figure 9:**
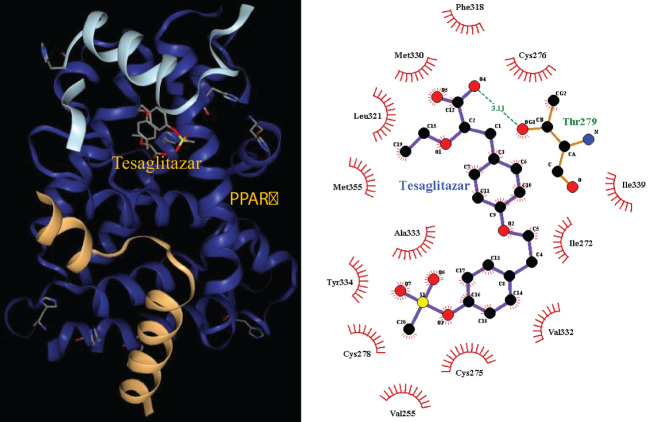
Molecular docking 3D map of PPARɑ ligand-binding domain with Tesaglitazar and 2D pose view of ligand and receptor interactions.

The pathways related to patient survival were submitted to the DSigDB database to find potential drugs. As a result, 7 related drugs were screened (**Table 1**). The PPAR signaling pathway was selected to further explore the possible drug action sites. The results show that PPARɑ Thr279 may form hydrogen bonds with Tesaglitazar (**Figure 9**).

**Table 1:** Drug mining based on the seven pathways related to patient survival.

KEGG pathway	Drug	Odds Ratio	Adjusted P-value	Role in T cell function
Cytosolic DNA sensing	Melitten	63	2E-9	CD4 T cell activation and proliferation (PMID: 33852824)
Antigen processing and presentation	Tanespimycin	103	3E-10	CD8 T cell exhaustion (PMID: 22949327)
Chronic myeloid leukemia	Nimbolide	302	5E-18	
PPAR signaling	Tesaglitazar	1141	2E-22	T cell survival, activation, and CD4 T helper cell differentiation (PMID: 22382683).
Primary immunodeficiency	Vincristine sulfate	47	3E-6	T cell development, proliferation, activation (PMID: 35091087)
Cardiac muscle contraction	Gabapentin	806	6E-34	
*Vibrio cholerae* infection	Vanadium	23	4E-5	

## Discussion

Sepsis is a heterogeneous disease, as diverse immune cell types are involved and many microorganisms can cause the disease. Current analysis of sepsis is mainly focused on single-source data. We integrated bulk transcriptome, single-cell transcriptome, and methylation data to find potential disease biomarkers and mechanisms. The integration of single-cell data is necessary as researchers cannot discriminate which cell produces the signal in bulk transcriptome data. Knowing the cell source of gene expression is necessary for the studies on mechanisms and treatment (18). Due to disease heterogeneity, the result of a single gene-based analysis sometimes lacks reproducibility (18). Therefore, we propose transcriptome analysis at the pathway level to identify differential pathways associated with sepsis. We analyzed pathways associated with survival, the relationship between pathways, immune cells, and the source of infection. We found T cell dysfunction in sepsis and used the T cell exhaustion-related pathway to mine related therapeutic drugs. This article provides useful ideas for the study of sepsis mechanisms, diagnosis, classification, and drug treatment.

Gene set-based analysis has been reported to yield more consistent results (19). This paper systematically analyzed the expression of the KEGG pathway in sepsis (**Table S1**). The identified differentially expressed pathways are supported by the literature. For example, cardiac dysfunction and hypotension are hallmarks of septic shock, which correspond to myocardial contractions and vascular smooth muscle contractions identified here (20). VEGF signaling is down-regulated in a rat model of sepsis, and this is consistent with our findings (21). In severe sepsis, protein breakdown occurs in skeletal muscle, leading to malnutrition in the body, as well as respiratory muscle dysfunction and pulmonary complications. This process is mainly involved in the ubiquitin-proteasome protein degradation pathway (22). This is consistent with the proteasome up-regulation identified here. Apoptosis, exhaustion, and function decline of T cells can lead to immunosuppression. Immune dysfunction is an important reason for the high mortality of sepsis. The acquired immune system dominated by T cells plays a key role in the late stage of sepsis (23). This is consistent with the down-regulation of the TCR signaling pathway found in this paper. LTP is the main molecular mechanism underlying learning and memory, and cognitive function and synaptic plasticity are decreased in septic mice (24). This is consistent with the LTP down-regulation found in this paper. Cytosolic free DNA as a danger signal is recognized by the DNA sensor cyclic GMP-AMP synthase (cGAS) and activates downstream signals to promote the production of type I interferons and other cytokines, thereby generating a corresponding immune response (25). We found that this pathway is up-regulated, which may be related to the inflammatory response. Furthermore, cluster analysis revealed intrinsic heterogeneity of the KEGG pathway in septic patients, suggesting the complexity of the septic disease. This may be caused by a variety of factors, such as the patient’s own genetic and physiopathological characteristics, differences in the period of infection, and different types of bacterial or viral infections. Therefore, it is difficult to carry out clinical diagnosis and treatment through a single molecule, but various factors should be considered comprehensively, and different treatment strategies should be formulated for patients with different characteristics.

This paper analyzed the relationship between differential KEGG pathways and survival, and verified the importance of differential pathways (**Figure 3**). Compared with healthy subjects, antigen processing and presentation are down-regulated in sepsis patients, but their expression is positively correlated with patient survival, suggesting that this pathway plays a protective role in sepsis. Antigen processing and presentation is an important component of acquired immunity and is involved in the recognition of pathogenic antigens (26). Sepsis is an important factor in the death of patients with CML, and the CML pathway is down-regulated in sepsis (27). Cytokine-cytokine receptor interaction pathway plays a dual role in sepsis. Moderate inflammation can fight off foreign pathogens, and excessive inflammatory response can lead to tissue damage, organ failure, and even death (28). *Leishmania* infection was considered SIRS, and its clinical manifestations and biochemical analysis are similar to those of sepsis (29). The PPAR signaling pathway acts on multiple links of the inflammatory signal transcription pathway, inhibits the inflammatory response, and thus has a certain protective effect on sepsis (30). The clinical features of primary immunodeficiency resemble severe sepsis, with severe cases associated with fever, shock, and multiple organ failure (31). Progesterone improves sepsis syndrome by reducing inflammatory cytokines, IL-6 and TNF-α and by restoring the antioxidant defense system. Therefore, progesterone may help control inflammation in sepsis, and this pathway is up-regulated in sepsis (32). *Vibrio vulnificus* sepsis has a high mortality rate (33). Therefore, related pathways can serve as potential therapeutic targets and prognostic signals.

This paper analyzes the relationship between the KEGG pathway and immune cell type (**Figure S1**). For example, CD4^+^ T cell abundance has the highest correlation with the TCR signaling pathway, which is positively correlated with 28-day survival. Neutrophils, a type of polymorphonuclear leukocytes, are the most abundant circulating leukocyte population in the human immune system, accounting for 50% to 70% of all circulating leukocytes in healthy adults. Neutropenia patients are more prone to microbial infection (34). We found that the number of neutrophils, which is highly positively correlated with the TLR signaling pathway, was positively correlated with survival. Neutrophil cell abundance was up-regulated in sepsis, suggesting its protective roles (**Figure S2**). We found macrophages were associated with survival, but with opposite directions in total and M0 or M1 macrophages, indicating that anti-inflammatory M2 macrophages may play a protective role (35). It has been reported that macrophage polarization can induce cytokine storm and immune paralysis, and macrophage activation can lead to early death in sepsis (36; (37). Therefore, these cells are potential cellular markers or targets for subtyping or risk assessment, such as the high abundance of certain immune cells with a lower risk of death (**Figure 4**).

We integrated multi-omics data to mine the potential molecular mechanisms of sepsis. We focused on the well-known TCR signaling pathway, which is down-regulated in sepsis (**Figure 5A**). Cell level analysis identified a down-regulated abundance of T cells (**Figure S2**). DNA methylation analysis revealed the differentially methylated genes were enriched with GTPase activity (**Figure S3**), which controls T cell activation (38). A recent study showed that bacteria can exploit eukaryotic Rho GTPase signaling cascades to promote invasion and proliferation within their host (39). Among our identified top differentially methylated genes, TPST1 can be induced by lipopolysaccharides (LPS) in macrophages (40). KCNJ15 was found to be involved in bacteria clearance in infection (41). The two genes were mainly expressed on monocyte/macrophage (data not shown), and we found they were hypomethylated and up-regulated in sepsis.

At present, no research has proposed a pathway that can distinguish different types of infection, and determining the type of infection is the prerequisite for formulating a reasonable clinical treatment plan. The multiple pathways identified in this paper have good performance in distinguishing bacterial, viral sepsis, and SIRS, and some of them are also prognostic for patient survival, such as primary immunodeficiency, antigen processing and presentation, etc (**Figure 7**). The latest study found that plasma tyrosine biosynthesis was significantly decreased in rats with acute lung injury in sepsis (42). While we found that this pathway is up-regulated in viral sepsis, it can be inferred that plasma tyrosine levels may serve as a promising molecular marker for distinguishing bacterial from viral sepsis. Viral sepsis can cause down-regulation of cardiac muscle contraction, while viral infections have long been associated with heart damage. These results suggest that clinicians should formulate differential diagnoses and treatment strategies for different subtypes/causes of sepsis to effectively improve the treatment effect (43).

Multi-omics analysis revealed the T cell dysfunction in sepsis. At the pathway level, we found the expression changes in pathways of TCR signaling and antigen processing and presentation. In single-cell analysis, we found that T cell exhaustion was present in sepsis. In methylation analysis, several T cell-related genes were altered, such as TNFSF8 and AKT3. In cell subtype level analysis, we found that gamma-delta T cells and Tregs were associated with bacterial and viral infections, and Tregs were associated with mixed infection. Indeed, publications have reported the associations between the gamma-delta T cells and sepsis severity (44), and the associations of Tregs and secondary infection (45). These results provide possible cellular targets for treatment.

Finally, we used the T cell-related KEGG pathway to mine potential drugs and provide important information for the clinical application of related drugs (**Table 1**). For example, Gabapentin is an analog of gamma-aminobutyric acid (GABA), which has antiepileptic and analgesic effects, but it has the effect of lowering blood pressure and heart rate, so it may not be suitable for sepsis patients (33). Tesaglitazar, a dual PPAR agonist with an affinity for PPARɑ/γ, can increase insulin sensitivity and is used in the treatment of type 2 diabetes. We found that insulin signaling pathway was down-regulated in sepsis. Studies demonstrated that intensive insulin therapy could combat insulin resistance and decrease morbidity and mortality by modulating the proliferation, apoptosis, differentiation, and immune functions of certain immune cells, especially monocytes/macrophages, neutrophils, and T cells associated with sepsis (46). While PPARγ is a potential therapeutic target for sepsis, which can regulate cell growth and differentiation, and repress the TGFβ1, which is known to play immune suppression roles in cancer microenvironment (47; (48). Tesaglitazar can reduce the inflammatory response and has been used for glycemic control in burn patients (49). Interestingly, we found that LTBP1 was hypomethylated in sepsis, which encodes an important protein for the TGF-β1 activity regulation (47). We found a positive correlation between LTBP1 and TGF-β1. Elevated TGF-β1 could block naïve T cell differentiation towards a Th1 effector phenotype, and promotes their conversion towards the Treg which suppresses antigen presenting functions of dendritic cells (47). We found Treg is an unfavorable predictor for sepsis survival (**Figure 4**). Interestingly, TGF-β was recently found to be a main driver of T cell exhaustion (50). We observed that exhausted T cells (TS3 subcluster) expressed high levels of TGF-β and CD14^+^ macrophage expressed high levels of TGF-β (**Figure 6E**), which may provide a clue for the treatment. A feature of the COVID-19 sepsis cytokine storm is an abnormally increased TGF-β activity (51). Various anti-TGF-β therapies should be explored in the future. While tesaglitazar treatment in the kidney results in a decrease in TGF-β1 mRNA (52). Therefore, tesaglitazar may not only alleviate inflammation but also abolish T cell suppression. Nimbolide is the main component of neem leaf extract, commonly used in cancer treatment. The latest study finds it protects against endotoxin-induced ARDS by suppressing the nitrosative-oxidative stress, inflammatory cytokines, and chemokines expression (53). ARDS is the most common fatal complication of sepsis, therefore, nimbolide can be a promising candidate for sepsis treatment. Melitten is the main component of bee venom, which can cause muscle contracture and increase blood pressure (54). A study found that melitten has toxicity and antibacterial effect on clinically extensively drug-resistant bacteria (55). However, safe dose levels of melittin could not exhibit antimicrobial activity, which hinders its application in clinical practice. Tanespimycin, a derivative of the antibiotic geldanamycin, is used in cancer therapy by inhibiting tumor Hsp90 expression. Interestingly, a study finds it prolongs survival, reduces inflammation, and reduces lung damage in mice with sepsis (56).

In conclusion, the work is the first to perform integrative analysis of multi-omics data and to globally characterize the expression changes of sepsis at the pathway level, providing molecular markers and targets for diagnosis, prognosis, and drug development.

## Data availability statement

The original contributions presented in the study are included in the article/**Supplementary Material**. Further inquiries can be directed to the corresponding author.

## Author contributions

QL and MS: Methodology, Investigation, Writing – Review and editing. QZ: Validation, Writing – Review and editing. YL: Methodology, Formal analysis, Visualization, Writing – Original draft. JX: visualization, Writing – Review and editing. HF: Methodology, Supervision, Resources. All authors contributed to the article and approved the submitted version.
