# Supplementary material for: Integrated analysis of multi-omics data reveals T cell exhaustion in sepsis
Source: Front Immunol. 2023 Apr 3;14:1110070. doi: 10.3389/fimmu.2023.1110070 (PMC10108839; doi:10.3389/fimmu.2023.1110070)
Supplement: Supplementary file 1 [file DataSheet_1.pdf]

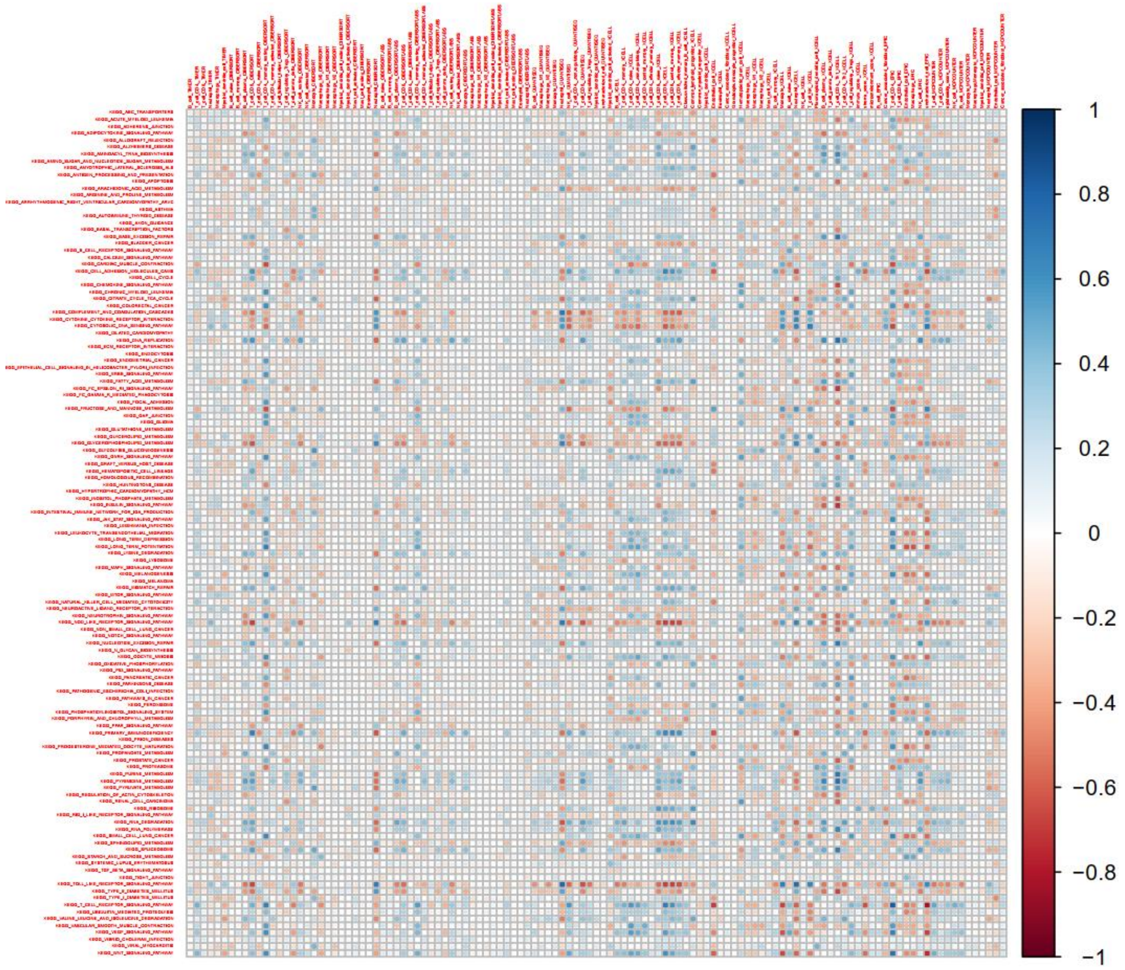

Fig. S1 The correlation plots show the correlation between the expression of KEGG pathways and the proportion of immune cells in sepsis

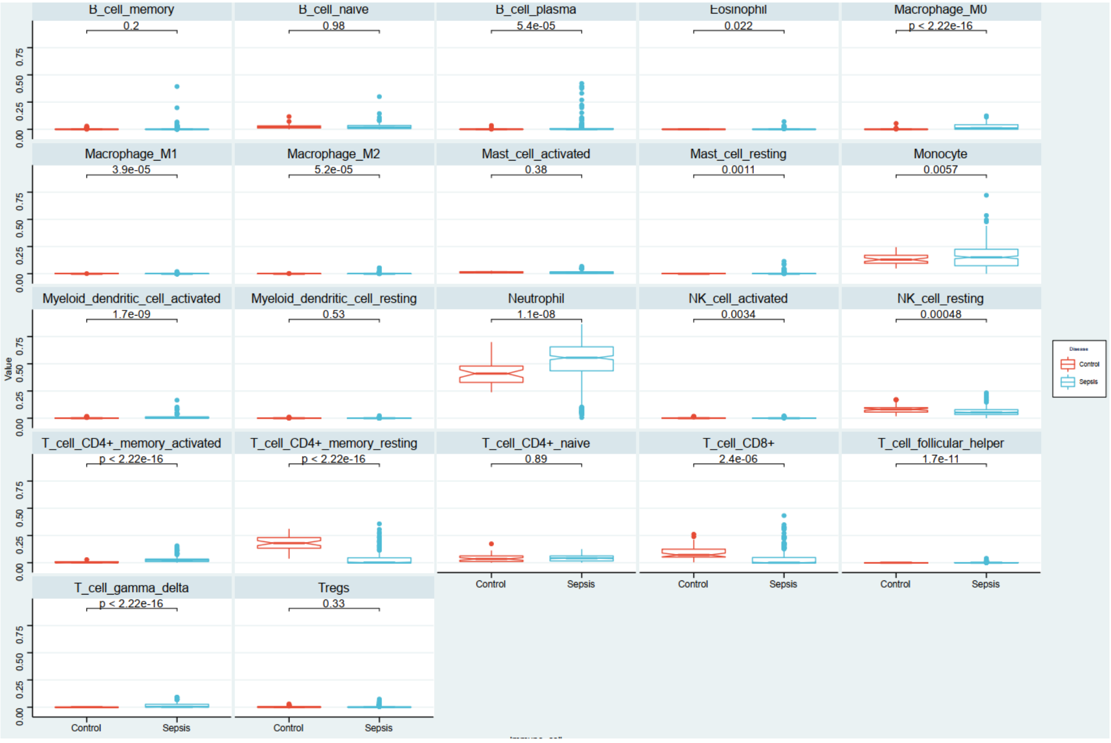

Fig. S2 Box plots showing the differential expression of twenty-two immune cells between control and sepsis

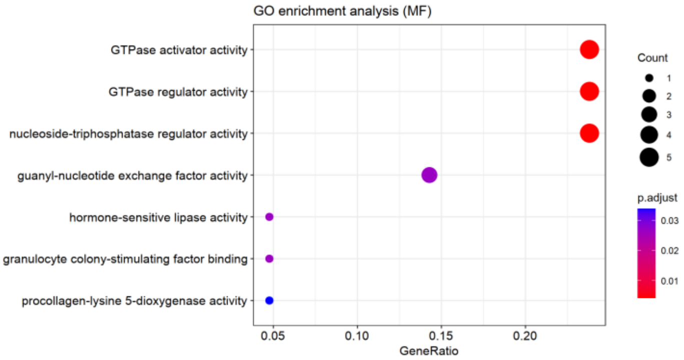

Fig. S3 The differential methylated genes were enriched with GTPase activity regulation in sepsis.

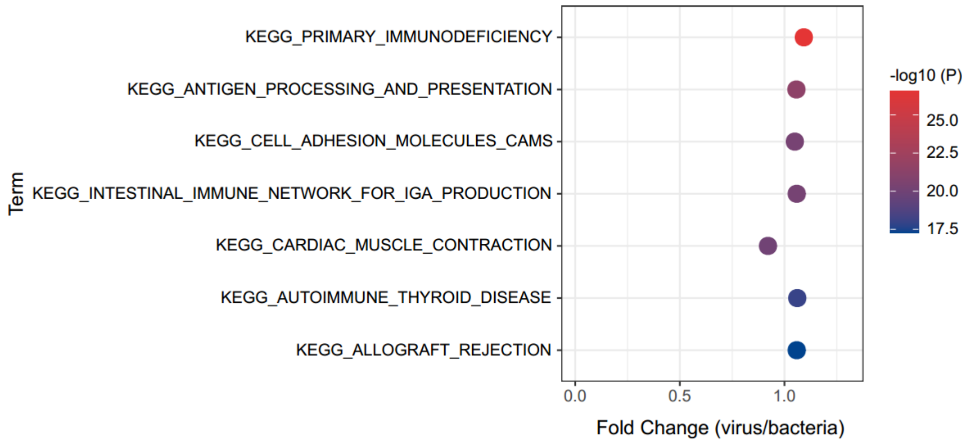

Fig. S4 The bubble plot of the differential KEGG pathways in viral/bacterial sepsis was analyzed in dataset GSE63990, showing the top 7 pathways with the most significant P values.

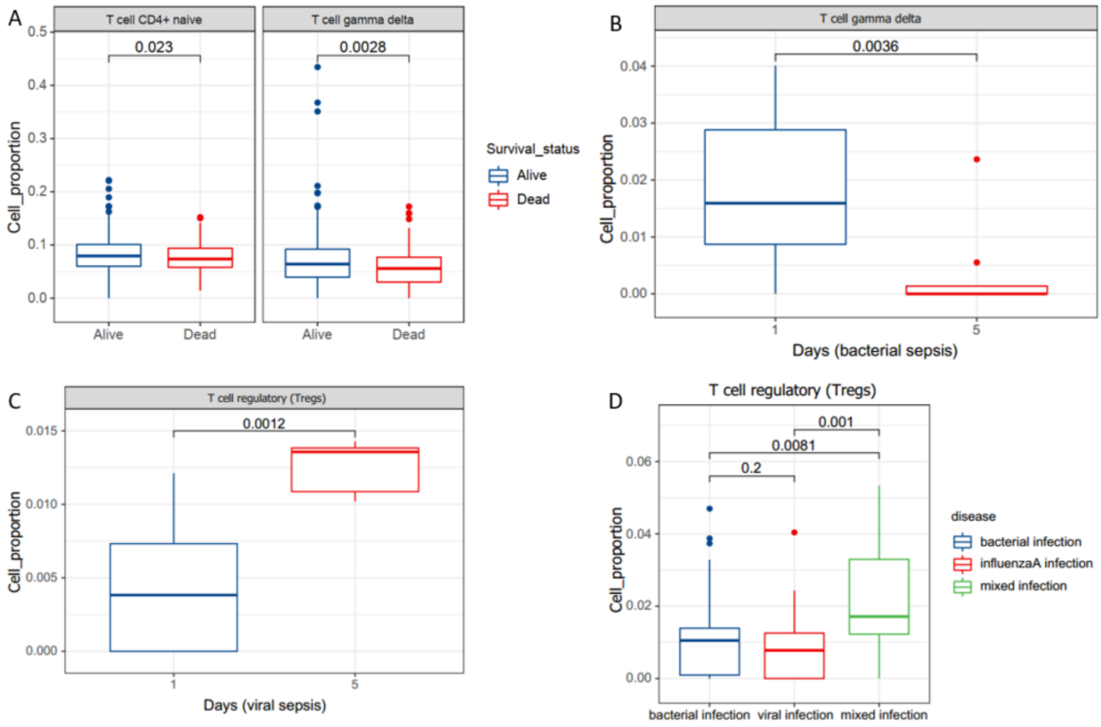

Fig. S5 Box plots showing the differential expression of T cell subsets in the septic process. (A) naïve CD4^{+} T cell and gamma-delta T cell were down-regulated in dead sepsis patients. (B) gamma-delta T cell was down-regulated in day 5 bacterial sepsis patients. (C) Tregs were up-regulated in day 5 viral sepsis patients. (D) Tregs were up-regulated in mixed infection patients compared to those bacterial or viral infection patients.
